# Effect of Different Physical Activity Training Methods on Overweight Adolescents

**Published:** 2010

**Authors:** Shohreh Ghatrehsamani, Noushin Khavarian, Maryam Beizaei, Reza Ramedan, Parinaz Poursafa, Roya Kelishadi

**Affiliations:** 1 Research Assistant, Isfahan Cardiovascular Research Center, Isfahan University of Medical Sciences, Isfahan, Iran; 2 Senior Expert, Isfahan Sport Training Organization, Isfahan, Iran; 3 Associate Professor of Pediatrics, Isfahan Cardiovascular Research Center, Isfahan University of Medical Sciences, Isfahan, Iran

**Keywords:** Children, Adolescents, Physical activity, Education, Obesity, Treatment

## Abstract

**BACKGROUND:**

In view of the growing trend of obesity around the world, including in our country, and the effect of reduced physical activity in increasing the incidence of obesity and overweight in children and adolescents and limitations of families in providing transport for their children to attend exercise classes, as well as time limitations of students in taking part in these classes, accessing appropriate methods for presenting physical activity training seems essential.

**METHODS:**

This non-pharmacological clinical trial was performed during six months from May to November 2007 on 105 children and adolescents aged 6-18 years with obesity, randomly assigned to 3 groups of thirty-five. Nutrition and treatment behavior were the same in all groups, but physical activity training in the first group was taking part in physical activity training classes twice a week, in the second group by providing a training CD, and in the third group via face-to-face training. Before and after the intervention, anthropometric indicators were measured and recorded.

**RESULTS:**

Mean body mass index (BMI) of participants in group attended physical activity training classes, and in the group undergone training with CD, after the interventions was significantly lower than that before the intervention.

**CONCLUSION:**

Our findings demonstrated that training using CDs can also be effective in reducing BMI in overweight and obese children and adolescents as much as face-to-face education and participation in physical training classes. Extending such interventions can be effective at the community level.

## Introduction

While until twenty years go, physical activity comprised a part of daily life of children and adolescents and they engaged in it as different games, walking, bicycling, and regular exercise, extensive studies in recent years have shown that due to changes caused by technological advances in social and environmental situation, the level of physical activity in many children and adolescents has decreased and is not at a level which can improve their health.^1^ Children and adolescents need physical activity more than other age groups, so that they can reach adequate physical growth and mental development and be able to fend off chronic diseases of old age. A sedentary lifestyle makes children and adolescents vulnerable in adulthood to chronic diseases, especially cardiovascular diseases and diabetes. Improving the health status in this vulnerable age group can be effective in prevention of epidemics of non-communicable diseases in developing countries.^2^–^4^ Among the negative consequences of reduced physical activity is the growing trend of the prevalence of overweight and obesity in the age group of children and adolescents during the past two decades, which of course are also due to changes in dietary habits, i.e., consumption of more fast foods and snacks.^5^ During the past two decades, the prevalence of obesity has increased, both in advanced nations and in developing and third world countries.^6^ For example, in England during the past two decades, the number of obese 6- and 15-year-old children and adolescents has doubled and trebled, respectively.^7^ The accelerated trend of overweight in children and adolescents should be considered as an alarming sign,^8^ because most of them will develop obesity in adulthood and obesity predisposes them to many chronic diseases (cardiovascular, diabetes, depression, etc.).^9^ In our country, overweight and obesity in children and adolescents has a growing trend and its relationship with lifestyle, including inactivity has been shown too.^10^ Some studies, through implementing regular training programs, such as holding training sessions at regular intervals and on a temporary basis, with various educational programs, including promoting self-confidence, physical activity training, modifying dietary habits and diet therapy, have been able to achieve useful outcomes in improving health indices in obese children and adolescents.^11^ Since various studies in different countries have shown that the effects of reduced physical activity in increasing the prevalence of obesity and overweight in children and adolescents are significantly more than the effects of changes in dietary habits,^12^–^14^ inclusion of physical activity training programs together with other programs (nutritional therapy and behavioral therapy) seems necessary. Then, achieving the best method of education can have the most prominent impact on controlling and treating obesity in children and adolescents.

## Materials and Methods

In this study, which was conducted as a non-pharmacological clinical trial for a period of six months (from 15 May to 15 November 2007), the target population consisted of 6 to 18-year-old children who were referred by physicians and schools to the Pediatrics Clinic of Isfahan Cardiovascular Research Center. They were randomly selected and placed in three groups. Sample size was calculated 105 in view of the quantitative nature of the variables at 105. Hence, 35 individuals were assigned to each group. The criteria for inclusion in the study were obesity and access to computer or video player (for film-based education). If the subject had thyroid diseases, hormonal abnormalities, mental retardation and severe physical disability that would hamper physical activities, the individual was excluded. Special recording files were created for all subjects and primary data including sex and age were recorded. Then, the required indicators were measured and recorded. All examinations were performed by the same team, which included a physician and a nurse. At the beginning, the weight of individual was measured by a scale in kilograms. The weight was measured in light clothing and without shoes. Then, height was measured in standing position next to a wall, with heels, hips, shoulders and head tangent to the wall. By dividing weight of individuals (kg) by square of height (m^2^), their body mass index was calculated and recorded. Waist circumference was measured from the point between the distance of 11^th^ and 12^th^ ribs to the anterior iliac spine in meters. Also, hip circumference was measured at the femoral trochanters and the ratio of the two was calculated and recorded as waist-hip ratio (WHR). Systolic and diastolic blood pressures were measured using a sphygmomanometer in mmHg. To make the study blind, the person who conducted measurements of indicators was not aware of the positioning of samples in the group in question.

In the first group, nutritional recommendations and behavioral therapy was performed in 10 sessions under supervision of the relevant expert in the form of monthly nutritional and behavioral treatment, and they took part in physical activity training classes twice a week. In the second group, nutritional recommendations and behavioral therapy was like the first group and the presentation of physical activity training was done by giving them educational CDs. In the third group, diet therapy and behavioral therapy was done as in the previous groups and physical activity training and nutritional education were done by face-to-face method. This program continued for 3 consecutive months and at the end of the third month, once more the indicators of each of the samples were evaluated and recorded. Analysis of data was performed by SPSS_15_ software. To compare the achieved changes between the three groups with different therapeutic methods, we used variance analysis test (ANOVA).

## Results

In this study, 105 children and adolescents in 3 groups of 35 were studied; their physical and general particulars are shown in Table 1. This table shows the primary data relating to each group. No significant difference existed among the three groups before intervention except in their WHRs. Comparison of variables under the study in three groups (the group participated in physical training classes, the group trained via CD, and the group undergone exercise recommendations) showed no significant difference in physical activity training with the three methods (Table 2). Table 3 shows mean and standard deviation of each of the variables in each of the educational groups before and after intervention. Mean height in all three groups before and after the intervention showed a significant difference (P < 0.001). Mean BMI in the group participated in physical activity classes and in the group undergone education withCD showed a significant reduction after intervention compared to those before intervention. Mean systolic blood pressure in the group participated in physical training classes and mean diastolic blood pressure in the group undergone education with CD showed a significant reduction.

**Table 1:** Mean (95%CI) level of indices studied in the three groups before intervention of study.

Indices	Groups			Total	P value

	Exercise class	CD	Exercise recommendation		
Weight (kg)	47.97 (43.51, 52.43)	46.35(40.78, 51.92)	47.80(42.80, 52.80)	47.38 (44.58, 50.17)	0.88
Height (cm)	143.11 (138.81, 147.41)	139.74 (134.33, 145.15)	141.97 (137.63, 146.30)	141.64 (138.99, 144.29)	0.57
Body mass index (kg/m^2^)	22.98 (21.77, 24.19)	23.07 (21.89, 24.24)	23.20 (21.88, 24.51)	23.08 (22.39, 23.76)	0.97
Waist circumference (cm)	80.76 (76.87, 84.64)	83.71 (79.87, 84.64)	81.72 (77.87, 85.58)	82.04 (79.83, 84.25)	0.54
Hip (cm) circumference	90.94 (87.38, 94.51)	89.77 (85.96, 93.59)	90.41 (86.23, 94.60)	90.39 (88.25, 92.52)	0.91
Waist to hip ratio	0.89 (0.86, 0.92)	0.93 (0.91, 0.95)	0.90 (0.89, 0.92)	0.91 (0.89, 0.92)	0.02
Systolic blood pressure (mmHg)	94.17 (90.38, 97.95)	99.70 (94.99, 104.41)	94.17 (89.59, 98.74)	96.01 (93.54, 98.48)	0.11
Diastolic blood pressure (mmHg)	54.86 (50.97, 58.75)	56.97 (52.84, 61.10)	53.83 (49.71, 57.96)	55.25 (52.98, 57.52)	0.54

**Table 2:** Mean (95%CI) level of indices studied in the three groups after intervention of study.

Indices	Groups			P value

	Exercise recommendation	CD	Exercise class	
Weight (kg)	47.14 (45.94, 48.33)	47.70 (46.45, 48.95)	46.34 (45.03, 47.45)	>0.05
Height (cm)	142.98 (142.56, 143.40)	143.56 (143.12 , 144.01)	143.06 (142.59, 143.52)	>0.05
Body mass index (kg/m^2^)	22.54 (22.02, 23.05)	22.63 (22.09, 23.16)	22.24 (21.68, 22.81)	>0.05
Waist circumference (cm)	82.48 (80.82, 84.13)	80.97 (79.26, 82.68	81.35 (79.59, 83.11)	>0.05
Hip (cm) circumference	90.16 (87.56, 92.76)	89.40 (86.72, 92.09)	92.11 (89.34, 94.89)	>0.05
Waist to hip ratio	0.91 (0.89, 0.92)	0.92 (0.90, 0.93)	0.89 (0.87 , 0.91)	>0.05
Systolic blood pressure (mmHg)	93.12 (89.60, 96.64)	93.67 (90.01, 97.33)	93.99 (90.20, 97.78)	>0.05
Diastolic blood pressure(mmHg)	53.99 (51.90, 56.09)	52.32 (52.52, 57.05)	54.79 (52.52, 57.05)	>0.05

**Table 3:** Mean (SD) indices before and after intervention in study groups

Indices	Groups	Mean difference before and after (0–3 month)	P value
Weight (kg)	Exercise class	−0.28 (1.76)	0.35
	CD	0.38 (1.47)	0.15
	Exercise recommendation	−1.07 (6.25)	0.36
Height (cm)	Exercise class	1.35 (1.11)	<0.001
	CD	1.91 (1.50)	<0.001
	Exercise recommendation	1.42 (1.18)	<0.001
Body Mass Index (kg/m^2^)	Exercise class	−0.53 (0.89)	<0.001
	CD	−0.45 (0.62)	<0.001
	Exercise recommendation	−0.85 (2.72)	0.09
Waist circumference (cm)	Exercise class	0.73 (6.21)	0.51
	CD	−1.45 (5.72)	0.17
	Exercise recommendation	−0.62 (3.57)	0.36
Hip circumference (cm)	Exercise class	−0.27 (2.88)	0.59
	CD	−0.94 (6.60)	0.44
	Exercise recommendation	1.72 (11.20)	0.41
	Exercise class	0.01 (0.07)	0.34
Waist to hip ratio	CD	−0.007 (0.05)	0.46
	Exercise recommendation	−0.02 (0.06)	0.18
Systolic blood pressure (mmHg)	Exercise class	−2.14 (4.89)	0.01
	CD	−3.64 (15.78)	0.19
	Exercise recommendation	−1.33 (10.66)	0.50
Diastolic blood pressure (mmHg)	Exercise class	−1 (6.95)	0.4
	CD	−1 (6.95)	0.05
	Exercise recommendation	0.17 (5.65)	0.87

## Discussion

Our findings showed that using the method of education with CD can also be as effective as face-to-face education and participating in physical training classes in reducing BMI of children and adolescents with overweight and obesity, and no significant difference was seen among the three methods of physical activity training.

Reduction of physical activity associated with changes in food and behavioral habits of children and adolescents has led to increase in overweight and obesity, turning into a serious problem in today's communities. In recent years, the amount of physical activity of our children and adolescents has been inadequate and although the participation of girls in exercise activities has been on the rise in recent years, it is still significantly less than that in boys.^15^ Other find-ings showed that nearly 60% of children and adolescents spend more than 4 hours in a day watching television^16^ whereas the maximum amount of time allowable for such sedentary activities is 2 hours daily.^17^ Given that increase in walking and reducing the amount of television watching has an important role in weight reduction in children,^18^ more attention must be paid to this matter. In a study conducted by Gorely and colleagues, 7-11 year-old children, in comparison with the control group, had increased physical activity after a 10-month intervention by physical training educational CD.^19^ As obesity can cause physical as well as mental disease in children and adolescents, it can also predispose them to chronic diseases of adulthood; therefore, the necessity of appropriate planning to weight control is of special importance. Individuals dealing with health of children and adolescents must use different methods to encourage families to improve their lifestyles and increase their children's physical activity, as well as their own. The important point about this matter is continued participation and physical activity of family members and emphasizing daily activities, rather than specialist and champion sports. In light of the fact that different studies have demonstrated the effect of reduced physical activity in increasing the prevalence of obesity of children and adolescents, even more than nutrition, it is necessary to integrate methods of physical activity training, including exercise accompanied by nutritional therapy and behavioral therapy. Therefore, achieving the best effects of educational methods for physical activity and also finding the most appropriate method of presenting this education can be a suitable solution in programs for control and treatment of obesity in children and adolescents. Hence the present study was designed with the aim of comparing three methods of physical activity training in this age group so that the obtained results can be extended to schools and the community. Given that parents are too busy to take their children to exercise classes and the time limitations of children and adolescents for participating in exercise classes, the use of educational methods such as CD made in this program can increase the level of physical activity in children and adolescents. It is recommended that wider studies be performed in the community so that the results can be further extended to the community.
